# Using natural language processing methods to classify use status of dietary supplements in clinical notes

**DOI:** 10.1186/s12911-018-0626-6

**Published:** 2018-07-23

**Authors:** Yadan Fan, Rui Zhang

**Affiliations:** 1 Institute for Health Informatics, University of Minnesota, Minneapolis, MN USA; 2 Department of Pharmaceutical Care & Health Systems, College of Pharmacy, University of Minnesota, Minneapolis, MN USA

**Keywords:** Natural language processing, Rule-based method, Machine learning-based classification, Dietary supplements, Use status, Clinical notes

## Abstract

**Background:**

Despite widespread use, the safety of dietary supplements is open to doubt due to the fact that they can interact with prescribed medications, leading to dangerous clinical outcomes. Electronic health records (EHRs) provide a potential way for active pharmacovigilance on dietary supplements since a fair amount of dietary supplement information, especially those on use status, can be found in clinical notes. Extracting such information is extremely significant for subsequent supplement safety research.

**Methods:**

In this study, we collected 2500 sentences for 25 commonly used dietary supplements and annotated into four classes: Continuing (C), Discontinued (D), Started (S) and Unclassified (U). Both rule-based and machine learning-based classifiers were developed on the same training set and evaluated using the hold-out test set. The performances of the two classifiers were also compared.

**Results:**

The rule-based classifier achieved F-measure of 0.90, 0.85, 0.90, and 0.86 in C, D, S, and U status, respectively. The optimal machine learning-based classifier (Maximum Entropy) achieved F-measure of 0.90, 0.92, 0.91 and 0.88 in C, D, S, and U status, respectively. The comparison result shows that the machine learning-based classifier has a better performance, which is more efficient and scalable especially when the sample size doubles.

**Conclusions:**

Machine learning-based classifier outperforms rule-based classifier in categorization of the use status of dietary supplements in clinical notes. Future work includes applying deep learning methods and developing a hybrid system to approach use status classification task.

## Background

The consumption of dietary supplements continues to grow worldwide. According to the most recent marketing data, Americans spent nearly $38.8 billion on dietary supplements in 2015 [1]. Due to the Dietary Supplement Health and Education Act (DSHEA) in 1994 [2], dietary supplements in the US markets are sold and regulated as a special category of food without safety testing before marketing. While dietary supplements are widely believed to be safe, they can cause adverse events, such as bleeding. A study conducted by Centers for Disease Control and Prevention (CDC) and Food and Drug Administration (FDA) estimated that 23,005 emergency department visits per year were attributed to the adverse events caused by dietary supplements [3]. Another major safety concern regarding the use of supplements is that prescribed drugs can interact with dietary supplements. The risk associated with drug-supplement interactions (DSIs) has gained increasing attention due to the widespread prevalence of dietary supplements in recent years, especially among the elderly, who are at greater risk for DSIs. Medications commonly prescribed among this population, such as anticoagulants and nonsteroidal anti-inflammatory drugs (NSAIDs), often tend to have serious interactions with dietary supplements, leading to dangerous clinical outcomes [4]. The bleeding induced by the interaction between warfarin and ginkgo is one example of DSIs [5].

The source information on supplement adverse events and DSIs mainly relies on voluntary reporting through post-marketing surveillance. Starting in 2006, dietary supplements companies were required by Dietary Supplements and Nonprescription Drug Consumer Protection Act to file reports of adverse events associated with dietary supplements to FDA [6]. However, the reporting is often inadequate and underestimated since such reporting is limited to severe adverse events, such as those leading to death, disability, and hospitalization. Moreover, there were very few clinical trials conducted to detect DSIs in the human population. Due to the inherent limitations of clinical trials such as sample size and limited study time, it’s often difficult to detect rare events. The lack of such information has posed a great risk to the health of the general population. To improve patient safety, it is imperative to increase our knowledge based on DSIs.

The data in the electronic health records (EHR), especially the clinical notes, serve as a great source for active pharmacovigilance on dietary supplements, as it captures longitudinal real word patient information on almost every aspect of clinical care, particularly those related to patient safety, including medication, laboratory results, signs and symptoms, etc. Similar to the medication information, clinical notes contain rich and valuable information on dietary supplements, especially the use status information. For example, there are mentions such as “She has started ginkgo for memory issue,” “Stop taking ginger before surgery,” and “The patient has discontinued taking ginseng two months ago.” Unlocking such information is critical for subsequent investigation on supplement safety research.

In the clinical domain, a number of studies have investigated the recognition of medication use status in clinical narratives through various methods including machine learning-based and rule-based methods. Pakhomov et al. [7] built a Maximum Entropy classifier along with a variety of different feature sets to categorize medication use status into four categories. Sohn et al. [8] used rule-based method and support vector machine (SVM) only with indication features to detect medication status change (e.g., no change, stop, start) in free text. Meystre et al. [9] performed prescription status classification on heart failure medications using SVM, reaching an accuracy score of 95.49% in the evaluation. Liu et al. [10] developed an SVM classifier using three types of features (i.e., contextual, semantic, discourse) to detect warfarin use status (ON or OFF) from clinical notes. Clinical notes have been extensively investigated to detect and recognize the medication use status. As for dietary supplements, we have previously investigated the detection of use status from clinical notes using both rule-based [11] and machine learning-based methods [12], and we also compared the performance of both classifiers based on a corpus with a smaller sample size of 1300 sentences [13].

In this study, we doubled the size of the corpus (2500 sentences) compared to our previous studies [13] and compared the performances. We tested more feature sets (e.g., bigrams, TF-IDF) with the supervised machine learning classification algorithms. We focused on 25 commonly used dietary supplements.

## Methods

### Data collection and annotation

The 25 commonly used dietary supplements were selected based on online consumer survey results [14], peer reviewed publications [15–17] and their availability in our patient cohort, which included alfalfa, biotin, black cohosh, Coenzyme Q10, cranberry, dandelion, Echinacea, fish oil, flax seed, folic acid, garlic, ginger, ginkgo, ginseng, glucosamine, glutamine, kava kava, lecithin, melatonin, milk thistle, saw palmetto, St John’s Wort, turmeric, valerian, and Vitamin E. Clinical notes mentioning the 25 supplements listed above were retrieved from clinical data repository (CDR) at the University of Minnesota. Institutional Review Board (IRB) approval was obtained to access the notes. A list of supplement names and their corresponding lexical variants generated by a pharmacist was used in the process of retrieving notes. For example, ginkgo and its lexical variants including ginko, gingko, and ginkoba were used. For each of the 25 dietary supplements, 100 sentences were randomly selected. A total of 2500 sentences were annotated following the annotation guideline in our previous study [11]. The use status of supplement in each sentence was given one of the four classes: Continuing (e.g., “Increase fish oil to 200 mg per day for high triglycerides”; “He was also continued on glutamine and Peridex to protect against mouth sores”), Discontinued (e.g., “The *Ginkgo biloba* was discontinued on admission”; “Pt did stop ginseng Oct 2013”), Started (e.g., “Continued joint pain, has added glucosamine and started exercise regimen last week”; “Patient is to start taking melatonin tonight to help her sleep”), and Unclassified (e.g., “She was advised to take NSAID’s PRN and Vitamin E daily”; “Recommend over the counter biotin 3 mg once daily”; “Pt inquiring about milk thistle”; “Avoid grapefruit, ginseng, and St. John’s wort”). Ten percent of the corpus was independently annotated by two raters with pharmaceutical background. Inter-annotator agreement was evaluated by Cohen’s kappa score (0.83) and percentage agreement (95%).

### Data preprocessing and splitting

The data was preprocessed as input to the classifiers. Preprocessing involved lowercasing as well as removing stop words, punctuations, and digits. Because of the time-constrained nature of the clinical setting, abbreviations are abundant in clinical documentation. Physicians often write abbreviations to improve efficiency and save time. For example, they often write “cont” to denote “continue”, “info” to denote “information”, “discontinue” has several forms of abbreviation, such as “dc”, “D/C”, “d/ced”. These abbreviations were replaced with their standard word form before normalization. All the sentences were then normalized using Lexical Variation Generation (LVG) [18]. The corpus was further split at the supplement level to generate the training and test datasets. Specifically, for each supplement, 100 sentences were randomly divided into two parts: 70 sentences (70%) for training and 30 sentences (30%) for test. In total, 1750 (70%) sentences out of 2500 served as the training data, and the remaining 750 (30%) sentences were used as test data.

### Development and evaluation of rule-based classifier

The rule-based classifier was developed on the training data and further tested using the test data. Two of our previous studies [12, 13] have shown that indicator words are extremely important in recognition and detection use status of dietary supplements. Based on the training data, a set of rules were generated using a variety of status indicators, which were compiled from reviewing the clinical notes and incorporated from other works identifying the use status of medications. Such indicator words included “start”, “restart”, “initiate”, “begin”, “add”, “resume”, “try”, “increase”, “decrease”, “continue”, “discontinue”, “stop”, “hold”, “off”, “recommend”, “advise”, “avoid”, etc. Some negated words were also included, like “no”, “not”, “never”, “decline”, “deny”. The indicator words were searched for within a window of the supplement mentions. We experimented with different window sizes starting from 0 to 11 tokens on both sides of the target words (supplement mentions). The best window size was selected based on the F-measure on the training data. The rules that were built from the training data were evaluated on the test data. Precision, recall, and F-measure were used as evaluation metrics.

### Development and evaluation of machine learning-based classifier

Compared with the previous study [13], more feature sets were trained when building the machine learning-based classifier, such as bigrams, the combination of unigrams, bigrams, and trigrams. Totally, five classification algorithms were trained along with nine types of feature sets on the training data. Five classification algorithms included decision tree, random forest, Naïve Bayes, Maximum Entropy and support vector machine (SVM). Nine types of feature sets were shown as follows: Type 1: raw unigrams without normalization; Type 2: unigrams (normalized); Type 3: TF-IDF (term frequency – inversed document frequency) for unigrams; Type 4: bigrams; Type 5: unigrams + bigrams; Type 6: unigrams + bigrams + trigrams; Type 7: indicator words only; Type 8: unigrams + bigrams + indicator words with distance (window size); Type 9: unigrams + bigrams + trigrams + indicator words with distance. We used 10-fold cross-validation to select the optimal parameters in the training data. All the trained models with optimal parameters were further evaluated on the test data. Precision, recall, and F-measure were used as evaluation metrics.

### Performance comparison

The performances of the rule-based and machine learning-based classifiers in terms of four use statuses in the test data were compared. Error analysis was conducted on the rule-based classifier to manually review the sentences that were falsely classified and identify the source of error. The precision, recall, and F-measure of the classifier with the best performance were further compared on each individual dietary supplement to evaluate the generalizability of the classifier across various dietary supplements.

## Results

### Dataset

In total, there were 604 sentences for C, 323 sentences for D, 425 sentences for S, 398 sentences for U in the training dataset. In the test dataset, there were 233 sentences for C, 166 sentences for D, 178 sentences for S, and 173 sentences for U.

### Performance of the rule-based classifier

**Fig. 1:**
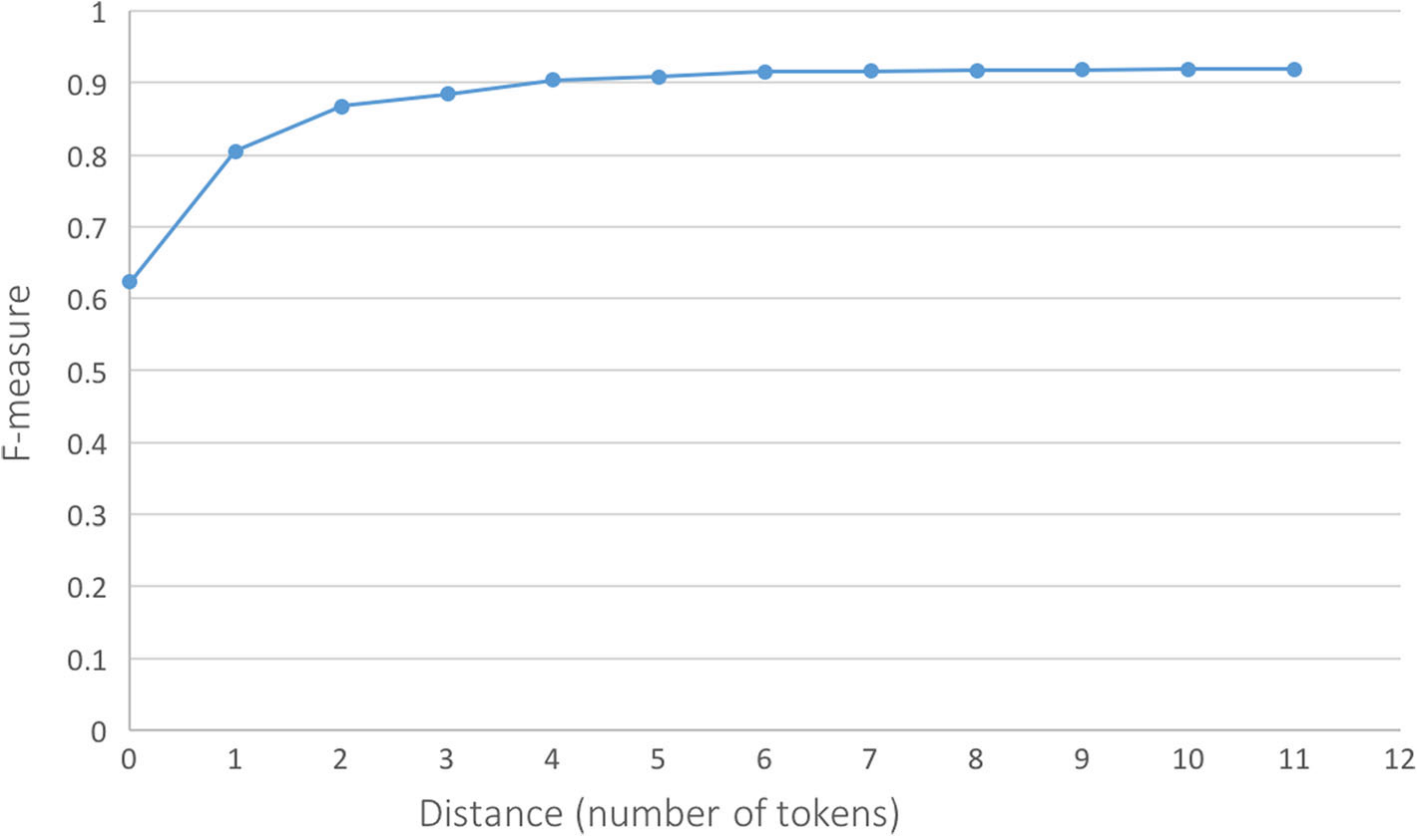
F-measures of the rule-based classifier with different window sizes on the training data

A total of 68 rules were generated. For each use status, the three most commonly used regular expressions and corresponding examples are shown in Table 1. The F-measure of the rule-based classifier with different window sizes in the training data are shown in Fig. 1. From the figure, we can see that F-measure increased sharply with the increasing distance and reached a stable state when the window size is 6 tokens. After 6 tokens, the performance went up very slowly with the enlarging distance. We arbitrarily set the window size to 7 in order to avoid over-fitting. The precision, recall, and F-measure for the four use statuses of the rule-based classifier on the test dataset are shown in Table 2. It shows that the F-measure for the four categories are all above 0.85, among which F-measures for continuing (C) and started (S) status are both 0.90.

**Table 1:** Selected rules and examples

Use status class	Frequently used regular expressions	Selected examples
Continuing (C)	continue(\s + \S+){0,7}\s + sup	Continue fish oil to reduce inflammation.
	increase(\s + \S+){0,7}\s + sup	She has increased her alfalfa tabs and this has eliminated her symptoms and chest tightness.
	take(\s + \S+){0,7}\s + sup	She is also taking a Vitamin E supplement and Tylenol as needed for pain.
Discontinued (D)	Stop(\s + \S+){0,7}\s + sup	Stop Vitamin E supplement.
	discontinue(\s + \S+){0,7}\s + sup	She is to discontinue her St. John’s Wort.
	hold(\s + \S+){0,7}\s + sup	You are already holding the fish oil and aspirin.
Started (S)	Start(\s + \S+){0.7} + sup	Started echinacea 1 week ago for cold.
	Add(\s + \S+){0,7} + sup	Add supplements with ginger.
	Begin(\s + \S+){0,7}\s + sup	I have asked him to begin using fish oil 3 capsules a per day, and he is agreeable to this.
Unclassified (U)	Recommend(\s + \S+){0,7}\s + sup	I did recommend taking over-the-counter fish oil, either 500 or 1000 mg per day.
	Avoid(\s + \S+){0,7}\s + sup	Avoid use of st. john’s wort on methadone as it can affect systemic level.
	Suggest(\s + \S+){0,7}\s + sup	Also suggested that she could consider trying otc Ginkgo biloba.

**Table 2:** Performance of the rule-based classifier (window size: 7) in the test data

Status	Number of sentences	Precision	Recall	F-measure
Continuing	233	0.90	0.91	0.90
Discontinued	166	0.92	0.80	0.85
Started	178	0.97	0.84	0.90
Unclassified	173	0.78	0.97	0.86
Total (weighted)	750	0.89	0.88	0.88

### Performance of the machine learning-based classifier

The performance of five machine learning-based algorithms with nine types of feature sets in the test data is shown in Table 3. As for the Type 8 and 9 feature sets, we experimented with different window sizes and selected the optimal window size as 6. The results showed that Maximum Entropy with type 8 or type 9 feature achieved the best performance. The precision, recall, and F-measure in terms of four use status of the optimal model are shown in Table 4. From the results we can see that the machine learning-based classifier achieved a satisfactory performance in terms of the four use statuses, particularly in C, D, and S, which have F-measures over 0.9.

**Table 3:** Performances of five classification algorithms with different feature sets in the test data

Type	Features	Decision tree			Random forest			Naïve Bayes			SVM			Maximum Entropy		
		P	R	F	P	R	F	P	R	F	P	R	F	P	R	F
Type 1	raw uni^a^	0.819	0.817	0.816	0.858	0.853	0.853	0.770	0.757	0.755	0.818	0.816	0.815	0.850	0.849	0.849
Type 2	uni	0.846	0.845	0.844	0.878	0.876	0.876	0.793	0.784	0.783	0.837	0.835	0.834	0.874	0.873	0.873
Type 3	tf-idf	0.862	0.857	0.857	0.862	0.857	0.857	0.763	0.704	0.701	0.844	0.839	0.839	0.840	0.831	0.831
Type 4	bi^a^	0.760	0.720	0.716	0.760	0.720	0.716	0.715	0.707	0.702	0.735	0.719	0.720	0.749	0.739	0.739
Type 5	uni + bi	0.872	0.864	0.863	0.872	0.864	0.863	0.815	0.808	0.807	0.881	0.877	0.876	0.890	0.888	0.887
Type 6	uni + bi+tri^a^	0.863	0.852	0.850	0.863	0.852	0.850	0.815	0.808	0.808	0.880	0.876	0.875	0.887	0.883	0.882
Type 7	indi^a^ only	0.848	0.847	0.846	0.861	0.860	0.860	0.860	0.849	0.848	0.851	0.849	0.849	0.862	0.859	0.859
Type 8	uni + bi+indi	0.860	0.860	0.860	0.875	0.865	0.864	0.813	0.803	0.801	0.899	0.897	0.897	0.895	0.903	**0.902**
Type 9	uni + bi+tri + indi	0.860	0.857	0.857	0.872	0.861	0.860	0.813	0.803	0.801	0.899	0.897	0.897	0.905	0.903	**0.902**

^a^uni: unigrams; bi: bigrams; tri: trigrams; indi: indicators

Bolded data represent the largest value

**Table 4:** The performance of Maximum Entropy with Type 8 feature set (unigrams + bigrams + indicators with window size of 6) in the test data

Status	Number	Precision	Recall	F-measure
Continuing	233	0.86	0.95	0.90
Discontinued	166	0.94	0.89	0.92
Started	178	0.92	0.91	0.91
Unclassified	173	0.92	0.84	0.88
Total (weighted)	750	0.91	0.90	0.90

### Error analysis of the rule-based classifier

We performed an error analysis for the rule-based classifier by manually reviewing the sentences that were incorrectly classified. In total, there were 89 sentences incorrectly classified by the rule-based classifier. As shown in Table 5, the source of error mainly consists of three parts: missing pattern issue, indicator words issue, and distance issue.

**Table 5:** Source of errors for the rule-based classifier

Source of error	Number of sentences	Percentage of errors
Missing pattern	47	6.3%
Indicator words issue	40	5.3%
Distance issue	2	0.3%
Total	89	11.9%

### Performance of machine learning-based classifier on dietary supplement level

The performance of the machine learning-based classifier on the individual dietary supplement in the test data is shown in Table 6. From the results in Table 6 we can see that for most dietary supplements, the F-measure is 0.9. For Vitamin E, the F-measure reached 1. However, the classifier has a poor performance on Coenzyme Q10 and milk thistle, the F-measures for which are below 0.8. Overall, our results demonstrated a good generalizability of the machine learning-based classifier for the majority of the dietary supplements.

**Table 6:** The performance of the machine learning-based classifier on the 25 dietary supplements in the test data

Dietary Supplement	Number	Precision	Recall	F-measure
Alfalfa	30	0.904	0.900	0.900
Biotin	30	0.927	0.900	0.904
Black cohosh	30	0.937	0.933	0.933
Coenzyme Q10	30	0.809	0.800	0.799
Cranberry	30	0.945	0.933	0.934
Dandelion	30	0.939	0.933	0.926
Echinacea	30	0.913	0.900	0.902
Fish oil	30	0.938	0.933	0.933
Flax seed	30	0.900	0.900	0.900
Folic acid	30	0.911	0.900	0.900
Garlic	30	0.919	0.900	0.903
Ginger	30	0.893	0.867	0.861
Ginkgo	30	0.943	0.933	0.932
Ginseng	30	0.947	0.933	0.935
Glucosamine	30	0.936	0.933	0.933
Glutamine	30	0.938	0.933	0.934
Kava kava	30	0.913	0.900	0.902
Lecithin	30	0.939	0.933	0.934
Melatonin	30	0.806	0.800	0.801
Milk thistle	30	0.787	0.767	0.751
Saw palmetto	30	0.907	0.900	0.900
St. John’s Wort	30	0.910	0.900	0.900
Turmeric	30	0.927	0.900	0.886
Valerian	30	0.944	0.933	0.928
Vitamin E	30	1.000	1.000	1.000

### Comparing the rule-based and machine learning-based classifiers

Comparing the performance of the two classifiers on the test data, the machine learning-based classifier achieved a better result with respect to the four use statuses, especially in the D status, whose F-measure improved from 0.85 to 0.92. For the C, S, and U status, the performance of the rule-based classifier is close to that of the machine learning-based classifier. Additionally, we also compared both classifiers in terms of the number of sentences which they both correctly classified, they both falsely classified, and only one of them correctly classified. From the detailed comparison results in Table 7, it indicates that the true positive rate in terms of C, D, and S status of the machine learning-based classifier exceeds that of the rule-based classifier. However, the rule-based classifier is more accurate in recall regarding U status.

**Table 7:** Comparison between the rule-based and the machine learning-based classifiers regarding four use statuses

Status	Number	TP^a^ for RB^a^	TP for ML^a^	RB (+)^a^ ML (+)	RB (+) ML (−)^a^	RB (−) ML (+)	RB (−) ML (−)
C	233	212	222	209	3	13	8
D	166	132	148	128	4	20	14
S	178	151	163	147	4	16	12
U	173	166	144	142	24	2	4

^a^TP: true positive; RB: rule-based classifier; ML: machine learning-based classifier; (+): correctly classified; (−): falsely classified

## Discussion

For all classification algorithms, normalized unigrams have a better performance compared with raw unigrams, indicating that normalization effectively reduces the feature space, thus improving the classification results. For some classification algorithms, such as decision tree and SVM, the TF-IDF features are more informative than unigrams, while for other algorithms, the performance degraded compared with unigrams. Bigrams are the least informative among the features sets, reflected by their poorest performance. Compared with only unigrams, the addition of bigrams and trigrams didn’t necessarily contribute to the improvement of the performance. For instance, for the random forest, the performance of Type 2 feature (unigrams) is better than that for Type 5 (unigrams + bigrams) and Type 6 (unigrams + bigrams + trigrams) feature sets. From the results of Type 7 (indicators only), we can see that indicator words hold significant information in use status. For example, for Naïve Bayes, the Type 7 feature set has the best performance compared with other feature sets. For decision tree, the Type 5 feature set (unigrams + bigrams) has the best performance. For random forest, the Type 2 feature set (unigrams) achieved the best result. For SVM, the Type 8 and Type 9 feature sets performed best. Among all the classification algorithms, Maximum Entropy with Type 8 or Type 9 feature sets achieved the same best performance (F-measure: 0.902).

Like the previous study [13], the sources of errors were mainly made up of three parts. First, there are new patterns we failed to generate from the training set. For example, “we reviewed her medications and cut out hyocyamine, biotin and scheduled the bentyl bid,” “Was taking milk thistle when he was living at home, but is no longer doing so,” “He denies taking any other GNC supplementation other than the ginseng and protein.” Second is the indicator issue: more than one use status indicator appears in the same sentence. For example, “Still off estrogen and started black cohosh because of nightsweats,” “He never stopped taking the saw palmetto,” “Pt quit taking turmeric – restarted less than a week ago.” Under such circumstance, the order of the rules largely impacts the performance of the rule-based classifier. Some errors are due to the indicator word being more than 7 tokens from the supplement mentions. From Table 5 we can see that the largest percentage of error mainly comes from “missing pattern” issue.

Our previous study [13] comparing the rule-based and machine learning-based classifier showed that the rule-based classifier is slightly better when the sample size is much smaller (1300 sentences). However, in the current study, the results indicate that the machine learning-based classifier is more accurate when the sample size (2500) nearly doubles. It should be noted that F-measure of U status of the machine learning-based classifier (F-measure: 0.88) is larger than that of the rule-based classifier (F-measure: 0.86), while in our previous study [13], the rule-based classifier (F-measure: 0.88) performs better in terms of U status than the machine learning-based classifier (F-measure: 0.77). Therefore, the results of the current study show that the performance of the machine learning-based classifier regarding U status not only has been greatly improved, but also outweighs the rule-based classifier.

It is evident that the performance of the rule-based classifier degrades when the sample size increases. The reason might be due to the fact that as the sample size increases, more patterns appear in both training and test datasets and the patterns generated by observing the training data cannot fully represent the test data. Another potential disadvantage of the rule-based classifier is that it is time-consuming and labor-intensive to develop the regular expression rules. In this respect, machine learning-based methods are more scalable and efficient.

The results of the machine learning-based classifiers (Table 3) show that the features are extremely significant in determining the performance of the supervised text classification algorithms. The performance varies with different feature sets. One limitation of this study is that we only tested 9 types of features. In the future, we will explore more types of feature sets and experiment with combinations of feature sets. Recently, there has been an increasing interest in applying deep learning methods to solve the text classification tasks. One major advantage of deep learning methods is that human-generated features are not required. In the future, we will attempt to try state-of-the-art deep learning methods, such as long short-term memory networks, to detect and classify the use status of dietary supplements from clinical notes. Our future work will also include making use of the specific advantages of both classifiers, such as high precision of the machine learning classifier and high sensitivity of the rule-based classifier to develop a hybrid system.

## Conclusions

In this study, both rule-based and machine learning-based classifiers were constructed to detect and categorize the use status of 25 commonly used dietary supplements into 4 use status classes. The performances of rule-based and machine learning-based classifiers were further evaluated and compared in the test data. The comparison results show that the machine learning-based classifier outperforms the rule-based classifier when the sample size increases to 2500 sentences. Future work includes applying deep learning methods and developing a hybrid system for identifying supplement use status in clinical notes.

## Abbreviations

C: Continuing
CDC: Centers for disease control and prevention
CDR: Clinical data repository
D: Discontinued
DSHEA: Dietary supplement health and education act
DSIs: Drug-supplement interactions
EHR: Electronic health records
FDA: Food and Drug Administration
IRB: Institutional review board
LVG: Lexical variant generation
NSAIDs: Anticoagulants and nonsteroidal anti-inflammatory drugs
S: Started
SVM: Support vector machine
TF-IDF: Term frequency - inverse document frequency
U: Unclassified
